# Identification of Two Dielectric Relaxations in Oleic-Rich Oils Within the 50–900 MHz Range Using a Low-Cost Method

**DOI:** 10.3390/foods15142460

**Published:** 2026-07-11

**Authors:** Inmaculada C. Fita, José M. Cruz

**Affiliations:** 1 Instituto Universitario de Investigación de Conservación y Mejora de la Agrodiversidad (COMAV), Universitat Politècnica de València, Camino de Vera s/n, 46022 Valencia, Spain; 2 Departamento de Física Aplicada (DFA), Universitat Politècnica de València, Camino de Vera s/n, 46022 Valencia, Spain; jmcruz@fis.upv.es

**Keywords:** reflection coefficient, NanoVNA, OECP, relative complex permittivity, Cole–Cole model, maxwell model, frequency relaxation, triglycerides

## Abstract

The dielectric spectrum of vegetable oils has been widely used to explore relationships with their physicochemical quality parameters. A Cole–Cole relaxation with a characteristic frequency around 200–300 MHz is commonly reported at 25 °C. More recently, the efficient heating of vegetable oils at 2450 MHz has been attributed to the relaxation of a glycerol component (10% by weight) in the triglycerides of vegetable oils. This study presents a method to identify more than one relaxation process using a NanoVNA directly connected to a 5-pin open-ended coaxial probe. The reflection coefficient S_11_(*f*) was measured and transformed into the relative complex permittivity ε(*f*) using the Marsland transformation. The resulting spectra were analyzed with the Cole–Cole model and with the Maxwell model incorporating two Debye relaxations. The method was validated over the 50–900 MHz range using silicone oil, glycerol, and pure triglycerides. The static dielectric constant was obtained with high accuracy. For the first time, two relaxations were observed in oleic oils, including a low-frequency relaxation between 86 and 96 MHz, close to the 100 MHz relaxation of glycerol at 25 °C. Additionally, the low-cost equipment and the open-source software make the method accessible to laboratories with limited resources.

## 1. Introduction

Vegetable oils are complex mixtures composed predominantly of triglycerides (glycerol triesters, >95%), in which the three hydroxyl groups of the glycerol backbone are esterified with fatty acids of varying chain length, typically ranging from C16 to C18. These fatty acids are commonly classified into saturated fatty acids (SFA), monounsaturated fatty acids (MUFA), and polyunsaturated fatty acids (PUFA) depending on the number of double bonds, generally from 0 to 3. In addition, vegetable oils contain minor components (<5%), including (i) healthy compounds as tocopherols, phytosterols, and phenolic compounds, and (ii) degradation products as diglycerides, free fatty acids (acidity index), phospholipids and peroxides (peroxide index). Specifically, the relatively high content of oleic acid (>55%, C18:1) and healthy compounds in extra virgin olive oil (EVOO) is highly valued for its potential cardiovascular benefits [1], whereas degradation products decrease oil quality [2]. All these parameters are evaluated by well-established physicochemical methods [3].

From a dielectric standpoint, triglycerides exhibit a low dielectric constant, owing to the permanent dipole of the ester group, the small dipoles associated with CH_2_ groups, and the number of double bonds in the order SFA < MUFA < PUFA [4]. Minor constituents may be classified as weakly polar compounds, such as sterols and tocopherols; polar compounds, such as phenolic compounds (present at low concentration, <0.04%); and highly polar degradation products.

In fresh vegetable oils, degradation compounds are present only in trace amounts; however, thermal processing leads to a reduction in healthy compounds and to an increase in degradation products.

The quality of thermally processed vegetable oils decreases with increasing levels of degraded polar compounds, which can be determined by silica gel column chromatography [5,6] and also by measuring dielectric permittivity using a specialized high-temperature capacitor [7,8]. A regulatory limit of 24% polar compounds has been established for heated oils [5]. Furthermore, a relationship between dielectric constant and polar compounds content has been reported [8].

In vegetable oils, the dielectric constant is close to three, reflecting the weighted contribution of their SFA, MUFA, and PUFA triglycerides, as well as their degradation products. Nevertheless, examining the decrease in permittivity with increasing frequency (relaxation) may help identify the compounds and molecular groups responsible for the dielectric behavior of oils.

In this regard, the search for direct relationships between physicochemical parameters and the variation in dielectric properties with frequency (*f*) through non-destructive procedures is of increasing importance, due to the higher speed, cost-effectiveness, and potential for on-site measurements [9,10].

In most published works on vegetable oils, dielectric measurements are only available from a few Hertz up to the 3 MHz range. In these studies, the main measured parameter was the static dielectric constant (ε_s_), which was related to: (i) the main fatty acid chains and their degree of unsaturation [11,12]; (ii) the appearance of polar species after applying thermal treatments [13,14,15,16]; (iii) the degree of adulteration in binary mixtures of various oils [17]; (iv) the free fatty acid content (FFA, %) and the degree of unsaturation, whereby ε_s_ decreased as both FFA (%) and the iodine value increased, and (v) the degree of oxidation, showing an increase in the ε_s_ value with the TOTOX index [18]. According to Lichi et al., the ε_s_ values of fatty acids increased with the number of double bonds or with molecular chain length. In vegetable oils, εs was mainly affected by unsaturated C18 fatty acids. Moisture under 0.10% did not affect ε_s_ results [11]. Only a few works have extended measurements to microwaves [19].

The dielectric spectra of vegetable oils, in the range 100 MHz–7 GHz, have predominantly been described in terms of the Cole–Cole dielectric relaxation model with parameters: static permittivity (ε_s,_ zero-frequency permittivity), high-frequency permittivity (close to ε_∞_), characteristic relaxation frequency (f_C_), and exponent α, which characterizes the symmetric broadening of the relaxation as a function of frequency, around f_C_. Some of these parameters have been reported as useful indices for distinguishing the origin of oils and assessing the quality of vegetable oils [14,20,21,22,23]. Values reported in these studies fall within the range of 240–340 MHz for olive oil [24,25].

In all these studies, the focus has been on determining the static dielectric permittivity and identifying a unique dielectric relaxation between 100 and 1000 MHz, for which no physical explanation has been provided. Reference data of ε_s_ and the Cole–Cole relaxation frequency are reported at 25 °C.

The dielectric behavior of vegetable oils around 2450 MHz has recently been reported, based on the unexpectedly rapid heating of these oils in microwave ovens compared with water and mineral oils [26,27]. The authors concluded that: “Glycerol component (10% by weight) in vegetable oils molecules is a major contributor to microwave heating”. This result prompts further investigation of the dielectric relaxations of vegetable oils at frequencies close to 100 MHz, the main relaxation of glycerol at room temperature [27].

Microwave heating promotes oxidation and the subsequent formation of polar compounds. However, unlike photooxidation and conductive (bulk) heating (thermoxidation), microwave heating selectively interacts with the functional groups that absorb energy, thereby enhancing oxidation as temperature increases [28,29].

Thus, the investigation of the dielectric relaxations of vegetable oils at 25 °C in the 50–900 MHz range is of considerable interest, since this interval includes the 100 MHz relaxation associated with glycerol and the single Cole–Cole relaxation frequency reported for vegetable oils, around 240–340 MHz.

In the literature on dielectric measurements up to the GHz range, two domains have been applied: (a) time domain reflectometry (TDR), a time-based measurement technique, implemented using TDR equipment [20,30], and (b) frequency-domain reflectometry (FDR), a frequency-based measurement technique, implemented using a vector network analyzer (VNA) with an open-ended coaxial probe (OECP) for reflection measurements [25,31]. Both methods are based on the measurement of the complex reflection parameter (S_11_(*f*)). To convert S_11_(*f*) into the relative complex dielectric permittivity (ε(*f*)), two main types of procedures have been used [32]: (i) those that require standard reference materials for calibration, such as the capacitive model (two or three calibration media) and the radiative antenna model (four reference media) [33]), and (ii) those that do not require calibration media, such as the inverse solution method, e.g., the point-matching theory [34], and the full-wave moment method [35].

In recent years, low-cost nano vector network analyzers (NanoVNA) operating up to the GHz range have been used with probes coupled to the end of a coaxial cable to measure S_11_(*f*) as the reflected wave, when the probe is immersed in the liquid under investigation. Several works described the use of NanoVNA for the determination of the broadband complex permittivity of porous media [36,37] and for the characterization of liquid foods [38]. The NanoVNA was able to achieve decent results in all measurements, with adequate accuracy in most cases. The dynamic range was satisfactory up to 1 GHz [39], with a low investment (less than 200 €).

For measuring S_11_(*f*) in liquid samples, custom-made probes of various types have been used. Among these, OECP using a semi-infinite sample configuration was the most widely employed [40]. Some configurations were based on an SMA connector whose pins were cut flush with the base, for measuring honey [41], others were employed with several 50 cm long pins for soil moisture estimation [30,42,43]. A key parameter of the OECP is the electric-field penetration depth, which depends on the permittivity of the sample and the measurement frequency. High frequencies and high permittivity values lead to a smaller electric-field penetration depth. For example, the penetration depth at 2450 MHz was reported to be about 6 m in silicone oil; greater than 200 mm in vegetable oils; about 16 mm in water; and 13 mm in glycerol [27]. Small electrode separations (<10 mm) were recommended in order to obtain a nearly uniform electric field, thus yielding measurements that are representative of the sample volume. Other probe types, such as resonant circuits, have been used for lipid oxidation monitoring, whose main advantages are low cost and on-site measurements [44,45].

The main objective of this study was to assess a simple and low-cost method, based on FDR measurements with a NanoVNA and an inexpensive commercial 5-pin probe (OECP-5p), for identifying two dielectric relaxations in four oleic-rich vegetable oils (74–84%) over the 50–900 MHz range at room temperature (25 °C). In this study, we first analyzed a single-relaxation Cole–Cole model to facilitate direct comparison with the existing literature, and we then considered a two-relaxation Maxwell model, which provides a clearer physical interpretation by identifying individual Debye relaxation frequencies.

## 2. Materials and Methods

### 2.1. Materials

#### 2.1.1. Oleic-Rich Vegetable Oils

Oleic-rich oils with long-chain fatty acids (LCT) and predominantly monounsaturated fatty acids (MUFA > 70%), mainly oleic acid, were selected.

The oleic-rich oils selected for this work were extra virgin olive oil (EVOO), olive pomace oil (OPO), and high-oleic sunflower oil (HOSO); hereafter, they will be referred to as oleic oils for simplicity.

The four oleic oils evaluated in this work were: early harvested EVOO from the 2025–2026 campaign in Valdeolivas, Cuenca, Spain (EVOO-1), EVOO from the 2024–2025 campaign in Jaen, Spain (EVOO-2), olive pomace oil (OPO) from the 2024–2025 campaign in Jaen, Spain, and high-oleic sunflower oil (HOSO) bottled in Seville, Spain. In Table 1, the profile of the principal fatty acids is shown. The moisture content of all oils was below 0.10%, as the oils were extracted using a two-phase decanter and subsequent filtration.

#### 2.1.2. Reference Dielectric and Chemical Compounds

Water for analysis ExpertQ, and Acetone HPLC grade of Scharlau (Barcelona, Spain) were used as reference materials in the Marsland transformation, and to validate the reliability and the accuracy of the proposed method: (1) silicon oil (polydimethylsiloxane, PDMS) 100% (Pequinsa labs, Pamplona, Spain), (2) glycerol 99.5% (Pequinsa, Pamplona, Spain), (3) tricaprylin (Caprylic Acid Triglyceride) a medium-chain triglyceride (C8:0 MCT) derived from coconut, which offers homogeneity as it contains exclusively caprylic acid 99%, and (4) triacetin (1,2,3-Triacetylglycerol) short-chain triglyceride (C2:0 SCT) with very short-chain acetic acid 99% (Sigma-Aldrich, Madrid, Spain).

Cleaning of the probe was done with 2-Propanol multisolvent and n-Hexane 99%, HPLC grade of Scharlau, both solvents.

### 2.2. Methods

The procedure for obtaining ε(f) was structured into three successive steps: (1) calibration of the NanoVNA, (2) measurement of S_11_(*f*), and (3) determination of ε(f) using the Marsland transformation with four reference materials, short, air, water and acetone.

To validate the proposed method, reference compounds with known dielectric properties were measured: (i) silicone oil, which exhibits no dielectric relaxation; (ii) glycerol; (iii) triacetin, a short-chain triglyceride (SCT), and (iv) tricaprylin, a medium-chain triglyceride (MCT). The dielectric relaxation models applied to ε(f) were: the single-relaxation symmetric Cole–Cole model, and the Maxwell model with two Debye-type relaxations, the latter for which no previous references exist in the characterization of vegetable oils. In addition, the electrical characterization of the OECP-5p (Clarkson theory) was done first for water and acetone, and then for oleic oils and triglycerides.

#### 2.2.1. Instrumentation

**Figure 1:**
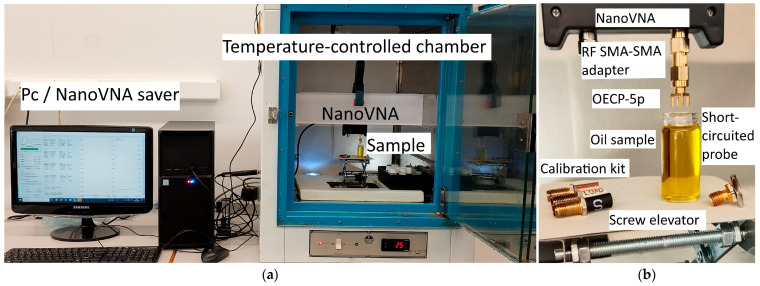
Experimental setup: (**a**) temperature-controlled chamber containing NanoVNA and oil sample, PC running NanoVNA Saver and (**b**) detail of the NanoVNA showing the SMA adapter to the 5-pin SMA-probe (OECP-5p). Oil sample, calibration kit, and short-circuited probe on the screw elevator.

The NanoVNA-H4, made by ZeenKo, was selected as a very tiny handheld and affordable device (Figure 1a). This NanoVNA is a two-port vector analyzer, with two-channel ports for reflection and transmission measurements. It works in the range from 50 kHz to 1.5 GHz. It was used to measure the reflection coefficient S_11_(*f*) through port 1, which is defined as:

(1) $$S_{11}\left(f\right)=\frac{\left[Z_{in}\left(f\right)-50\right]}{\left[Z_{in}\left(f\right)+50\right]}$$

S_11_(*f*) reveals the properties of the material at the end of the transmission line where the probe is immersed, with Z_in_(*f*) being the input impedance of the material surrounding the probe, and 50 Ω being the real (resistive) impedance of the line between the VNA and the probe.

S_11_(*f*) measurements were performed using an OECP of five pins connected to a NanoVNA. The central pin of the probe is surrounded by four external pins symmetrically separated from each other at a radial distance of 3 mm from the central pin (Figure 1b). Pins are 4 mm in length. External pins are interconnected and tied to the system ground. This commercial probe with 5 pins (OECP-5p) offers the following advantages: (i) high capacitance, (ii) a highly uniform electric field, (iii) excellent symmetry and pin alignment, (iv) small sample volume, and (v) lower thermal inertia, enabling more rapid attainment of thermal equilibrium. The OECP-5p is well-suited for measuring low dielectric constant within the 50 kHz to 900 MHz frequency range. The main characteristics of this OECP-5p are: nominal impedance 50 Ω, frequency range 0–18 GHz, v.s.w.r = 1.05 and RF insertion loss = 0.05 at 1 GHz according to the technical data sheet [46]. An RS Pro Straight RF adapter SMA plug to SMA plug (18 GHz) was connected between port 1 of NanoVNA and the probe. Both the adapter and the OECP-5p are covered with a thin gold layer to avoid brass oxidation and loss in conductivity. The probe was tightened to the adapter with a torque wrench to keep them together with the same force in every measurement.

The NanoVNA was interfaced with the PC through USB and registered via open-source software GitHUB NanoVNA Saver 0.5.5.0 [47]. Sweep settings were selected from 50 kHz to 900 MHz. Sweep averaging was used, which allowed discarding of outlying samples to improve the average. The number of measurements to average was 9, and the number to discard was 4. Sweep control was configured in two segments, obtaining 4.47 MHz/step and 201 points of S_11_(*f*).

#### 2.2.2. Calibration and Measurement of S_11_(*f*)

Calibration procedure was done in the VNA-probe plane with a calibration kit (short, open and 50 Ω load) before every five measurements set as recommended by the manufacturer to avoid systematic errors.

The frequency window for FDR measurements was set to 50–900 MHz. This range was chosen as a compromise between ensuring reliable instrument performance and preserving comparability with reference triglycerides, whose reported relaxation processes require relatively wider frequency windows than vegetable oils. Extending the range beyond 900 MHz up to the instrument limit (1500 MHz) was avoided because previous measurements on reference liquids showed increased dispersion above 900 MHz, which would reduce the robustness of the fitted dielectric parameters.

For each material, three independent samples were collected, and each sample was measured five times, resulting in 15 measurements per material. Measurements were taken inside a controlled temperature chamber at 25 °C (Figure 1a). Maintaining the measurement equipment, the probe, and all materials inside the chamber at a constant temperature was essential to ensure measurement reproducibility, since the permittivity decreases with increasing temperature [11,12]. A screw elevator was used to transform the samples and submerge the probe up to the base of the pins (Figure 1b).

#### 2.2.3. Clarkson Antenna Radiative Theory

To characterize the OECP-5p, the theoretical transformation of [48], as used by [30] in the characterization of a 7-wire probe, was employed. The complex reflection parameter S_11_(*f*) is:

(2) $$S_{11}\left(f\right)=\frac{A+e^{-2\gamma L}}{1+A\cdot e^{-2\gamma L}}$$

being the complex factors A and γ:

(3) $$A=A\left[\varepsilon \left(f\right),f\right]=\frac{1-z\sqrt{\varepsilon (f)}}{1+z\sqrt{\varepsilon (f)}}$$

(4) $$\gamma =\gamma \left[\varepsilon \left(f\right),f\right]=\frac{2\pi f\sqrt{\varepsilon (f)}}{c}j$$

where L and z are the characteristic parameters of the OECP-5p: L is the probe length, and z = Z_c/_Z_pa_, where Z_pa_ is the probe impedance in air, and Z_c_ is the characteristic impedance of the cable connected to the probe (measured at the VNA calibration plane). j is the imaginary unit (j^2^ = −1), c is the speed of electromagnetic waves in vacuum (c = 3·10^8^ m/s).

ε(*f*) is the complex relative permittivity of the medium surrounding the probe in a given measurement:

(5) $$\varepsilon \left(f\right)=\frac{Complex\;Dielectric\;Permittivity}{\varepsilon_{0}}=Re\left(\varepsilon \right)+j\cdot Im(\varepsilon )$$

where ε_0_ = 8.854·10^−12^ (C^2^/(N·m^2^)) is the vacuum permittivity. Re(ε) is the dielectric constant, and the absolute value of Im(ε) is the dielectric loss due to the absorption and dissipation of the electromagnetic energy associated with the dielectric relaxation processes. It is worth mentioning that for triglycerides and vegetable oils, the dc electrical conductivity that produces the dissipation of energy associated with ionic and charge transport is negligible.

The OECP-5p was characterized using reference liquids spanning a wide range of dielectric constants, including high- and medium-permittivity solvents such as water and acetone (Section 3.1.2), as well as low-permittivity standards such as silicone oil (Section 3.3). In addition, the probe was characterized using oleic oils and triglycerides as described in Section 3.3.

#### 2.2.4. Transformation of S_11_(f) into ε(f)

A flexible open-source software library (Version 2021) for estimating the complex permittivity, PyOECP [49], was used to transform the measured reflection coefficient S_11_(*f*) to ε(*f*), based on the antenna model proposed by Marsland and Evans [33].

The model consists of an equivalent electrical circuit with three elements in parallel: (i) a capacitance associated with the dielectric material in the neck of the probe, (ii) another capacitance through the sample between the pins of the probe, and (iii) a conductance due to radiation [31,50,51,52]. The derived expression for the permittivity of unknown material (ε_U_) is:

(6) $$G\cdot {\left(\varepsilon_{U}\right)}^{5/2}+\varepsilon_{U}+\frac{D_{42}\cdot D_{13}}{D_{41}\cdot D_{32}}\left(\varepsilon_{3}+G\cdot \varepsilon_{3}^{5/2}\right)+\frac{D_{43}\cdot D_{21}}{D_{41}\cdot D_{32}}\cdot \left(\varepsilon_{2}+G\cdot \varepsilon_{2}^{5/2}\right)=0$$

where G is the radiation term:

(7) $$G=\frac{-D_{41}\cdot D_{32}\cdot \varepsilon_{4}+D_{42}\cdot D_{13}\cdot \varepsilon_{3}+D_{43}\cdot D_{21}\cdot \varepsilon_{2}}{D_{41}\cdot D_{32}\cdot \varepsilon_{4}^{5/2}+D_{42}\cdot D_{13}\cdot \varepsilon_{3}^{5/2}+D_{43}\cdot D_{21}\cdot \varepsilon_{2}^{5/2}}$$

where ε_1_, ε_2_, ε_3_, and ε_4_ are the relative dielectric permittivity of four reference materials (short-circuited probe, air, water, and acetone, respectively) measured at the probe–medium plane and D_ik_ = S_11_(i) − S_11_(k) for i, k = 1, 2, 3 and 4. The first reference is a short-circuited probe [S_11_(short) = −1 + 0j with ε_1_ = ∞], constructed with an OEPC-5p and a metal welded to the external pins (Figure 1b). The second reference is air [S_11_(air) = 1 + 0j with ε_2_ = 1 + 0j]. Determination of ε_U_ requires that the implicit Equation 6 be numerically solved by iteration.

The PyOECP software (version 2021) includes functionality for random noise reduction using the Savitzky–Golay smoothing filter for signal processing and recommends a default window length of 81. However, based on our previous experience with reference materials (methanol and ethanol), we observed that smoothing altered the parameters of the reference model. Excessive filtering distorted the true spectral shape, particularly in the vicinity of the loss peaks, which are critical for the accurate determination of relaxation times. Therefore, the minimum window length (set to 5) was selected to better preserve the raw permittivity values, which were subsequently fitted to the proposed electrical models.

#### 2.2.5. Electrical Models of Dielectric Relaxations

- Cole–Cole model (one relaxation)

**Figure 2:**
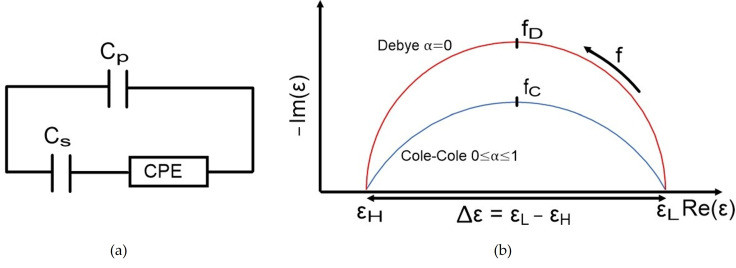
(**a**) Equivalent circuit of one dielectric relaxation; (**b**) dielectric permittivity plot for Cole–Cole and Debye models.

Cole–Cole equivalent circuit (Figure 2a) is the reference electrical model used in the bibliography for representing one dielectric relaxation in vegetable oils. This circuit represents a symmetric dielectric relaxation (Figure 2b) between the permittivity at high frequency ε_H_ (C_p_) and the permittivity at low frequency ε_L_ (C_p_ + C_s_).

CPE is a constant phase element which represents the frequency-dependent relaxation of permittivity, with Y_0_ and α as characteristic parameters, whose admittance is:

Y(CPE) = Y_0_·(jω) ^α^ and the equivalent capacitance is: C(CPE) = Y(CPE)/(jω) = Y_0_·(jω)^α−1^.

Therefore, the complex relative permittivity associated with CPE is:

(8) $$\varepsilon \left(CPE\right)=\frac{Y_{0}}{\varepsilon_{0}}{\left(j\omega \right)}^{\left(\alpha -1\right)}$$

being ω = 2·π·*f*. The exponent α indicates the relaxation shape in the permittivity plot (Figure 2b).

If α = 0, the plot appears as a semicircle (Debye or single relaxation), but if 0 < α ≤ 1, the plot is represented by a flattened semicircle (Cole–Cole relaxation). These circuit elements define the real characteristic parameters of the relaxation: (i) high-frequency permittivity (ε_H_ = C_p/_ε_0_), (ii) permittivity increment Δε = C_s/_ε_0_, and (iii) low-frequency permittivity: ε_L_ = Δε + ε_H_.

The characteristic frequency of the symmetric relaxation is the frequency for the maximum absolute value of Im(ε(*f*)) and the highest variation in Re(ε(*f*)), which is given by:

(9) $$f_{C}=\frac{1}{2\pi }{\left(\frac{Y_{0}}{C_{s}}\right)}^{\left(\frac{1}{\left(1-\alpha \right)}\right)}$$

The general relative complex dielectric permittivity equation (Davidson–Cole), for a single relaxation, with τ = (2π·f_C_)^−1^, results in:

(10) $$\varepsilon (f)=\varepsilon_{H}+\frac{\varepsilon_{L}-\varepsilon_{H}}{{\left[1+{\left(j\omega \tau \right)}^{\left(1-\alpha \right)}\right]}^{\beta }}$$

Equation (8) represents: (i) a symmetric single Debye relaxation, if β = 1 and α = 0, (ii) a symmetric Cole–Cole relaxation, if β = 1 and 0 < α ≤ 1, and (iii) an asymmetric Davidson–Cole relaxation, if 0 < β ≤ 1 and α = 0. If a Cole–Cole relaxation presents values of exponent α (larger than 0.2), it can be inferred that there is the presence of more than one Debye-type dielectric relaxation. The Maxwell model was used to model two single Debye relaxations.

- Two-relaxation Maxwell model

**Figure 3:**
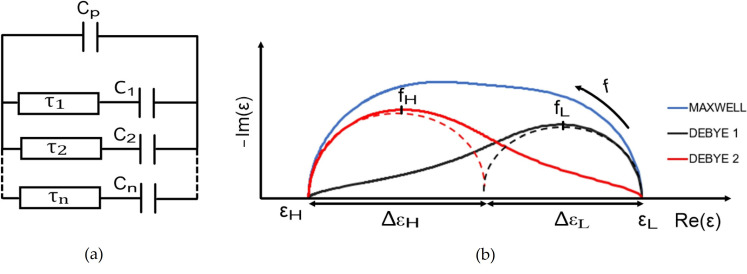
(**a**) Equivalent circuit of Maxwell model with n relaxations: C_p_, $\tau_{i}$ and C_i_ are the circuit elements, and (**b**) dielectric permittivity plot for the Maxwell model of two relaxations: imaginary part − Im(ε) versus the real part Re(ε) of the two single Debye contributions to the permittivity and the resultant permittivity sum of them, shown as solid lines. Permittivity plot for each individual relaxation, considered separately, plotted as dashed lines (semicircles).

Maxwell equivalent circuit (Figure 3a) is the reference electrical model used for representing various single dielectric relaxation [53]. Every branch of the circuit (Figure 3a) represents a single Debye relaxation; the higher the number of branches, the higher the number of Debye relaxations with characteristic frequencies, f_i_ = (2π· $\;\tau_{i}$ ) ^−1^ and dielectric increment Δε_i_ = C_i/_ε_0._ Capacitances C_i_, C_p_ and time constant $\tau_{i}$, denote the elements obtained by fitting the experimental data to the Maxwell equivalent circuit.

The total dielectric increment for the two relaxations, Δε_L_ in the low-frequency region and Δε_H_ in the high-frequency region, is given by:

(11) $$\varepsilon_{L}-\varepsilon_{H}=\Delta \varepsilon_{L}+∆\varepsilon_{H}$$

The general equation for the relative complex dielectric permittivity with two relaxations is:

(12) $$\varepsilon (f)=\varepsilon_{H}+\frac{\Delta \varepsilon_{L}}{1+j\cdot \omega \cdot \tau_{L}}+\frac{\Delta \varepsilon_{H}}{1+j\cdot \omega \cdot \tau_{H}}$$

The dielectric plot for two relaxations is in Figure 3b. The characteristic relaxation frequencies are calculated as: f_L_ = (2π· $\tau_{L}$ )^−1^ and f_H_ = (2π· $\tau_{H}$ )^−1^.

#### 2.2.6. CNLS Fitting and Statistical Analysis

Complex nonlinear least squares (CNLS) of the open access LEVM program [54] was applied to fit relative permittivity data to different equivalent circuits based on a model of one relaxation (Cole–Cole) or a model of more than one relaxation (Maxwell). The CNLS fitting, supported by its statistical goodness-of-fit indicators, reliably confirms the existence of two relaxations and rules out three or more additional relaxations. The two-relaxation Maxwell method can identify two closely spaced Debye relaxations with physical meaning, whose contributions to the imaginary part of the permittivity dominate within the measurement range. Therefore, relaxations outside the frequency window would contribute to a lesser extent and would not be detected.

To ensure robust convergence and to avoid local minima in the CNLS fitting of the experimental permittivity data, a sequential multi-step strategy was employed prior to the final result. This transformation was particularly necessary because the imaginary part of the complex permittivity Im(ε) was several orders of magnitude smaller than the real part Re(ε), making the fitting process highly sensitive to the choice of weighting and to the presence of noise in the smaller component. In the first step, an initial fit was performed using modulus weighting. This transformation treats the real and imaginary parts separately, minimizing the sum of their squared residuals with weights derived from the modulus of the data. The estimated parameters obtained from the modulus weighting fit were then used as starting values for a second fit with proportional data weighting. Finally, these refined values served as the input for the main fitting configuration: a single, comprehensive minimization combining function proportional weighting with an iterative reweighting scheme and simultaneous fitting of both permittivity components [55,56].

The LEVM analysis yields the parameters for each circuit element along with their associated uncertainties (p ± u(p)), as well as the global parameter (PDRMS), which indicates the percentage root-mean-square value of the relative residuals of all free parameters of the fit, and the estimated standard deviation of the weighted residuals (SF), a measured of the overall goodness-of-fit, that represents the maximum percentage deviation between the model and the measured data [57].

All experiments were performed independently in triplicate, and five replicates were measured for each sample. Since the fifteen dielectric spectra were fitted to the dielectric models, the results of these parameters are presented as average ± standard deviation (SD) (n = 15). Significant differences among groups were evaluated by one-way analysis of variance (ANOVA), followed by Student–Newman–Keuls post hoc analysis for multiple comparisons of average values. All statistical analyses were performed using Statgraphics version 19 (x64). Differences were considered statistically significant at *p* < 0.05.

## 3. Results

### 3.1. Measurements of the Complex S_11_(f)

In this section, the measured reflection coefficient S_11_(*f*) (real and imaginary parts) is presented as a function of frequency over the range, 63 MHz to 864 MHz, plotted on a logarithmic frequency scale. It is worth noting that, although the measurement range was 50 kHz–900 MHz, the analysis was performed over a narrower frequency interval to reduce the data dispersion observed at the edges of the range.

#### 3.1.1. S_11_(f) of Reference Materials for Marsland Transformation

**Figure 4:**
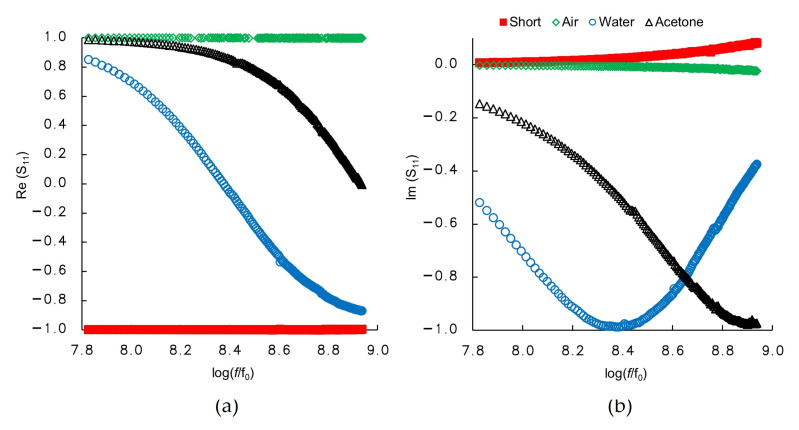
Reflection coefficient S_11_(*f*) measurements for reference materials: short-circuited probe, air, water, and acetone, on a logarithmic scale (log(*f*/f_0_)). (**a**) Real part of S_11_, (**b**) imaginary part of S_11_. Frequency (*f*) is expressed in Hz and reference frequency of f_0_ = 1 Hz, making (*f*/f_0_) dimensionless.

The S_11_(*f*) measurements of the reference materials, probe short-circuited and probe immersed in air, water, and acetone, required for the Marsland transformation are presented in Figure 4.

Re(S_11_) takes its constant value of +1 when the sample impedance is very large, Z_in_ = ∞ (Equation (1)), with Im(S_11_) = 0. In this case, total reflection occurs without a phase change, and all incident power is reflected. As shown in Figure 4, this situation occurred when the probe was surrounded by air. Re(S_11_) takes its constant value of −1 when Z_in_ = 0, with all incident power again reflected but with a phase reversal, and Im(S_11_) = 0. This condition is also observed in Figure 4 when the probe was short-circuited. Re(S_11_) for water and acetone exhibits intermediate, gradually decreasing values, transitioning from positive to negative, and asymptotically approaching −1 in the case of water.

Im(S_11_) is negative for both water and acetone, reaching a minimum value of −1. This minimum was reached at 242 MHz for water and at approximately 792 MHz for acetone. At these frequencies, values of Im(S_11_) = −1 with Re(S_11_) = 0 indicate that the OECP-5p, when filled with water or acetone, exhibited a purely capacitive input impedance of Z_in_ = −50j (Ω).

#### 3.1.2. Characterization of the OECP-5p for Reference Materials, Water and Acetone

To assess the consistency of the S_11_(*f*) measurements in water and acetone, the Clarkson radiative antenna theory was applied to estimate S_11_(*f*) for both materials (Equation (2)). Their dielectric permittivities are known [58,59].

Using the Clarkson theory, the estimated S_11_(*f*) was obtained. By minimizing the difference between the estimated and experimental S_11_(*f*), summed over all measured frequencies, the characteristic parameters of the OECP-5p: L and z, were determined. For water and acetone, values of L around 4 mm were obtained, which coincided with the length of the pins [30,43].

With L fixed at 4 mm, the parameter z was obtained by fitting the experimental S_11_(*f*) data to the estimated S_11_(*f*) values. When the sum of squared differences for Re(S_11_) and Im(S_11_) was minimized together, the resulting z values were 0.619 for water and 0.622 for acetone. The agreement between estimated and experimental values, obtained with L = 4 mm and z = 0.619–0.622, indicates that the S_11_(*f*) measurements correctly reflected the permittivity of water and acetone through antenna performance of the OECP-5p.

#### 3.1.3. S_11_(f) for Oleic Oils and Reference Dielectric Materials

**Figure 5:**
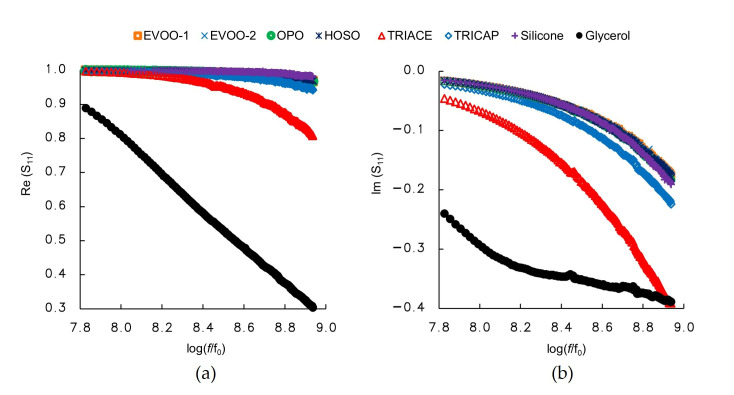
Reflection coefficient S_11_(*f*) measurements for one sample of: oleic oils: extra virgin olive oils (EVOO-1 and EVOO-2), olive pomace oil (OPO), triacetin (TRIACE), tricaprylin (TRICAP), silicone, and glycerol on a logarithmic scale (log(*f*/f_0_)). (**a**) Real part of S_11_ and (**b**) imaginary part of S_11_. Frequency (*f*) is expressed in Hz and reference frequency of f_0_ = 1 Hz, making (*f*/f_0_) dimensionless.

In Figure 5, the measured S_11_(*f*) values (real and imaginary parts) for the oleic oils, silicone oil, glycerol, tricaprylin, and triacetin are depicted.

For these materials, Re(S_11_) lies within the interval (0, +1). Re(S_11_) never reaches zero, which would occur only if Z_in_ = 50 Ω; therefore, Z_in_ > Z_0_ = 50 Ω for all the samples. For pure dielectrics, such as those tested here, lower values of Re(S_11_) correspond to higher values of the real part of the permittivity (relative dielectric constant). According to the data in Figure 5a, the dielectric constant of the measured materials is expected to be in the following order: silicone oil < oleic oils < tricaprylin < triacetin < glycerol. Im(S_11_) values range from zero to −0.40, decreasing progressively with frequency, which indicates dielectric materials with losses (Im(ε) < 0) and increasing loss level with the magnitude of Im(S_11_). In the measured frequency range, Im(S_11_) of oleic oils, triglycerides, silicone and glycerol does not reach −1 (Figure 5b), although this limit was attained by the reference materials, water and acetone (Figure 4).

### 3.2. Complex Relative Dielectric Permittivity of Reference and Measured Materials

**Figure 6:**
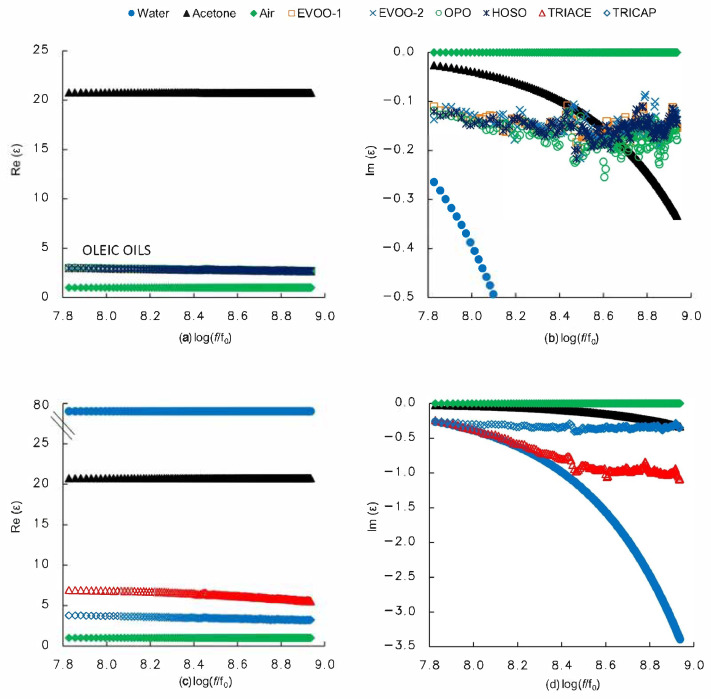
Relative permittivity for theoretical reference materials (air, water, and acetone) compared with experimental samples: oleic oils: extra olive oils (EVOO-1 and EVOO-2), olive pomace oil (OPO), and high-oleic sunflower oil (HOSO), and triglycerides: tricaprylin (TRICAP) and triacetin (TRIACE) on a logarithmic scale (log(*f*/f_0_)). (**a**) Real part of the permittivity and (**b**) imaginary part of the permittivity for oleic oils; (**c**) real part and (**d**) imaginary part of the permittivity for triglycerides. Frequency (*f*) is expressed in Hz and reference frequency of f_0_ = 1 Hz, making (*f*/f_0_) dimensionless.

Figure 6 presents the known permittivity values of the reference materials: air, water and acetone. For comparison, the permittivities obtained using the Marsland transformation are shown for oleic oils, triacetin, and tricaprylin. The short circuit is not plotted, as it is considered with infinite permittivity.

The values of Re(ε) for the measured triglycerides and oleic oils lie between those of the reference materials, acetone and air, being outside the range of acetone–water. The values of Im(ε) for the oleic oils lie between those of water and acetone from 7.6 to 8.6, and between those of acetone and air from 8.6 to 9, both in log scale of frequency (Figure 6a). The values of Im(ε) for triacetin and tricaprylin lie between those of acetone and water over the entire frequency range (Figure 6b). This result confirms that air, water and acetone are necessary and suitable as reference materials for the transformation of S_11_(*f*) into ε(f) in the study of the oleic oils and the triglycerides.

**Figure 7:**
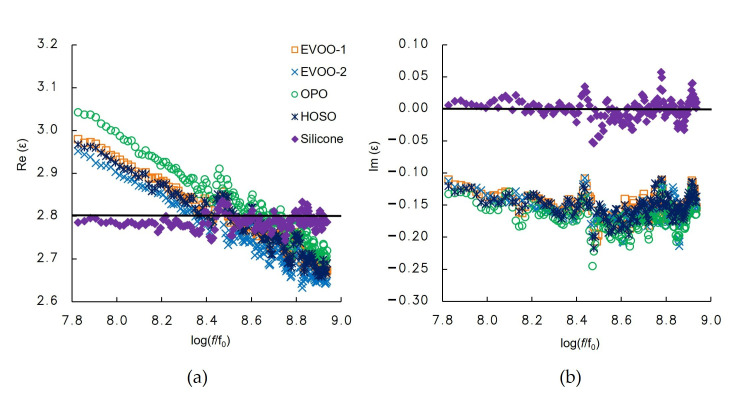
Relative permittivity of silicone oil compared with a single measurement of each oleic oil: extra virgin olive oils (EVOO-1 and EVOO-2), olive pomace oil (OPO), and high-oleic sunflower oil (HOSO). (**a**) Real part of permittivity, and (**b**) imaginary part of permittivity. Logarithmic scale (log(*f*/f_0_)), with frequency in Hz and reference frequency, f_0_ = 1 Hz, making (*f*/f_0_) dimensionless. The lines at Re(ε) = 2.8 and Im(ε) = 0.00 are included for reference only.

In Figure 7, the permittivity of the oleic oils and silicone oil is shown, for the purpose of comparison. This graph shows negligible dielectric losses (Im(ε) = 0) and a value of Re(ε) ≈ 2.8, for silicone oil, which is consistent with the literature data, reported in the range 2.60–2.75, [60,61,62]. Silicone oil is a mineral oil that exhibits no dielectric relaxation (zero imaginary component) and has a nearly constant dielectric constant, similar in magnitude to that of the oleic oils. This result validates the proposed method for the measurement and the Marsland transformation for oleic oils, demonstrating its ability to detect a negligible variation in Im(ε) for silicone oil and to distinguish it from the small relaxation observed in the oleic oils.

The oleic oils, EVOO-1 and EVOO-2, OPO and HOSO, display nearly linear relationships for Re(ε) in the range 2.65–3.10, while their imaginary component Im(ε) lies between −0.10 and −0.25, with the largest magnitude near the center of the interval (Figure 7b).

**Figure 8:**
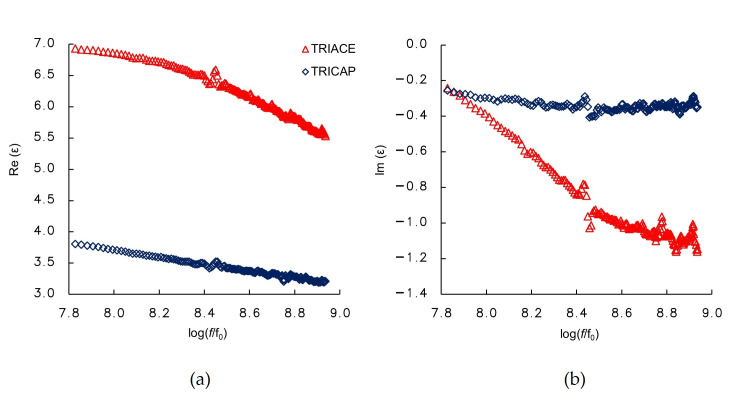
Relative permittivity for representative measurements for triacetin (TRIACE) and tricaprylin (TRICAP). (**a**) Real part of permittivity, and (**b**) imaginary part of permittivity. Logarithmic scale (log(*f*/f_0_)), with frequency in Hz and reference frequency, f_0_ = 1 Hz, making (*f*/f_0_) dimensionless.

In Figure 8, tricaprylin shows a higher Re(ε), ranging from 3.2 to 3.9, and a higher absolute Im(ε), lying between −0.25 and −0.40. Triacetin exhibits the highest values of Re(ε), ranging between 5.5 and 7.0, as well as the largest absolute values of Im(ε), between −0.2 and −1.2. The similar frequency-dependent behavior of tricaprylin and oleic oils (linear variation in Re(ε), and similar change in Im(ε) indicates a relaxation around 300–400 MHz (log(f/f_0_) = 8.6)), which is reasonable for the MCT and LCT, which make these materials weakly polar substances. However, triacetin is SCT, making it a polar substance, with higher Re(ε) values and nonlinear behavior (against log(f/f_0_)), along with a relaxation at a higher frequency in the imaginary part (the complete relaxation is not observed).

**Figure 9:**
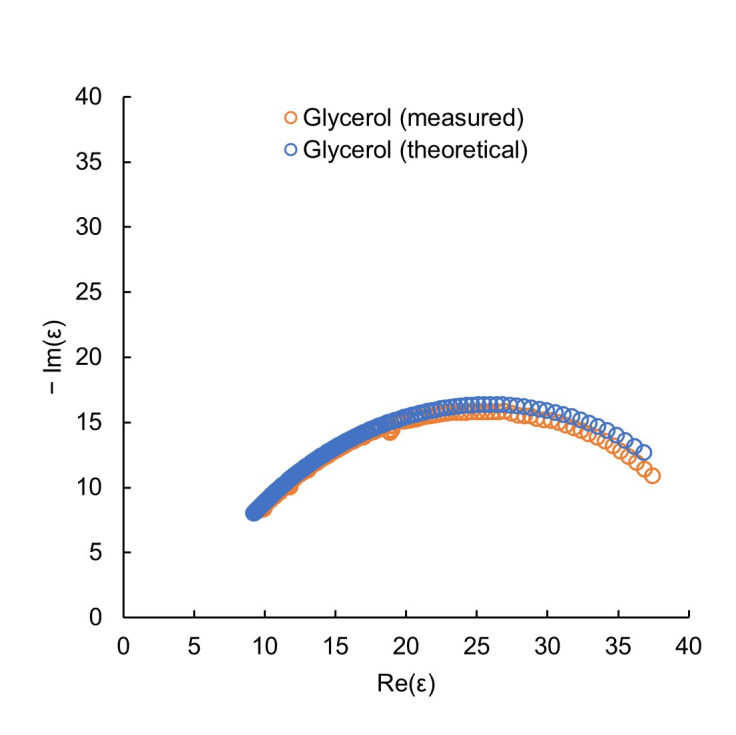
Permittivity plot of glycerol, measured in this work, compared with theoretical values based on the Davidson–Cole model as described by Kaatze et al. [63].

In Figure 9, the permittivity plot (Im(ε) vs. Re(ε)) of glycerol obtained by the proposed method is shown, together with the reference values from the Davidson–Cole model [63]. Some discrepancies are observed at low frequencies, at higher values of Re(ε), before reaching the peak of the absolute value of Im(ε). Nevertheless, the relaxation frequency of the measurement was around 114 MHz and agreed with the relaxation parameters reported by [63], over the range 400 kHz–3 GHz (ε_L_ = 43, τ = 1400 ps, β = 0.69 and ε_H_ = 4 in Equation (8)). It is worth noting that this relaxation frequency is not the frequency at which the magnitude of Im(ε) reaches the peak of the permittivity plot (about 152 MHz) for both curves, measured and theoretical, because of the characteristic asymmetry of the dielectric plot of glycerol, which is analyzed as the Davidson–Cole model.

These measurements with glycerol validated the proposed measurement–Marsland transformation for detecting the relaxation frequency in the interval 63–864 MHz. According to the authors [26,27], this relaxation of glycerol is responsible for the microwave heating of vegetable oils, which suggests that a relaxation near 100 MHz could also appear in the spectra of oleic oils at an ambient temperature of 25 °C.

### 3.3. Verification of the OECP-5p Performance as an Antenna for Oils and Triglycerides

As was done in Section 3.1.2 on the evaluation of S_11_(*f*) with water and acetone, the permittivity obtained from the Marsland transformation for different materials could be validated by comparing the measured S_11_(*f*) with the estimated S_11_(*f*) using Equation (2) with L = 4 mm. For oleic oils and triglycerides, the parameter z was estimated by least-squares fitting of the experimental values to the estimated ones, separately for Re(S_11_) and Im(S_11_).

The results of this analysis are listed in Table 2. Although all linear fittings between the estimated and measured values of S_11_(*f*), separated into their real and imaginary parts, were good for all the analyzed materials, the results for Im(S_11_) were considerably better than those for Re(S_11_), with R^2^ > 0.999, a slope closer to 1.0, and an almost zero intercept. This indicates that (i) the probe behaved as an antenna in the measurements of the imaginary part of S_11_(*f*), and (ii) the dielectric permittivities measured by the proposed method were acceptable mainly due to the good agreement in Re(S_11_) and excellent agreement in Im(S_11_).

The values of z obtained through Im(S_11_), estimated from their theoretical permittivities, were the highest, with 0.635 and 0.625 for water and acetone, respectively. For vegetable oils, z was obtained in the range 0.466–0.473; for silicone oil, 0.505; and for tricaprylin, 0.495, all falling into a similar range for weakly polar oils. For triacetin, *z* increased up to 0.561. The value of z increases with the static dielectric constant of the compound (Re(ε) at f = 0), from 0.466 to 0.495 when the permittivity is around 3–4, up to 0.561 at a permittivity of seven, and to 0.625–0.635 for higher permittivities, corresponding to acetone and water, respectively.

### 3.4. Dielectric Relaxation Models: Cole–Cole and Maxwell

**Figure 10:**
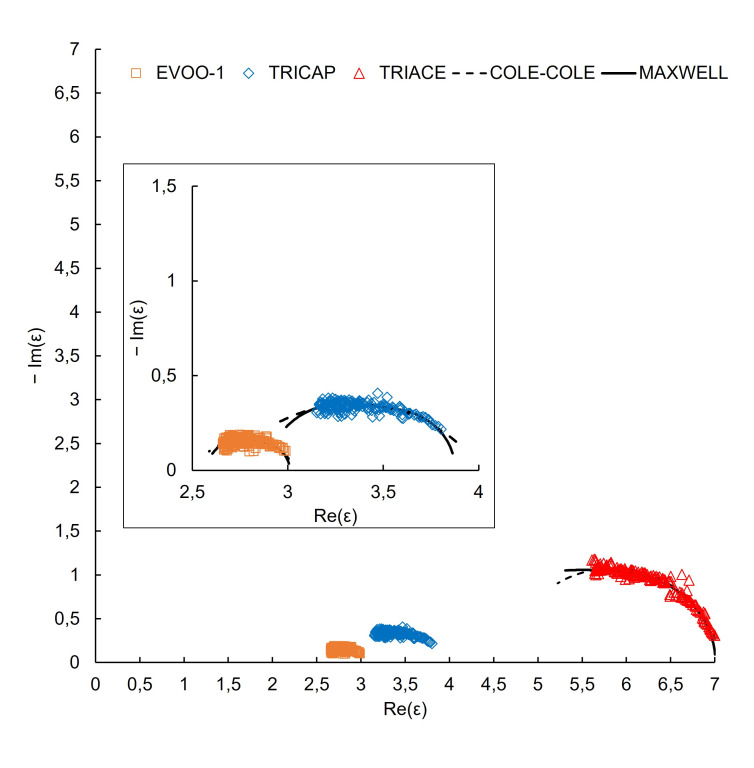
Permittivity plot and fitting to Cole–Cole and Maxwell models of one single measurement as an example, for the extra virgin olive oil (EVOO-1), and for triglycerides: tricaprylin (TRICAP), and triacetin (TRIACE). Inset for EVOO-1 and TRICAP.

The permittivity spectra, both real and imaginary parts, over the frequency range of 62–864 MHz for EVOO-1 (an example of an oleic oil), tricaprylin, and triacetin, for one replicate, are shown in Figure 10 as dielectric plots. The measured and fitted models for each material indicate that the models capture the behavior of the experimental data, with some differences between them. Each of the fifteen replicates for every oleic oil and triglyceride was fitted separately with the Cole–Cole and Maxwell models.

The goodness-of-fit was assessed using two complementary metrics: the percentage root-mean-square of the relative residuals (PDRMS) and the standard deviation of the weighted residuals (SF). Their interval values of fifteen fittings are shown in Table 3 and Table 4. The fits of both models were acceptable, with uncertainties below 6% for the individual parameters, PDRMS values between 2% and 24%, and SF values between 3% and 9% for the Cole–Cole model; and with uncertainties below 5% for the individual parameters, PDRMS values between 3% and 10%, and SF values between 3% and 9% for the Maxwell model.

The Cole–Cole equivalent circuit has four independent parameters: Y_0_, α, C_p_ and C_s_, while the Maxwell model has five independent parameters: C_p_, $\tau_{1},C_{1},\tau_{2},C_{2}$. The average values and standard deviation of the constant parameters characterizing the relaxations of both models are presented in Table 3 and Table 4, respectively.

Analysis of variance ANOVA was performed to assess the effect of oil type on the dielectric parameters, and the detailed results of this analysis are provided in the Supplementary Materials (Tables S1 and S2). For those parameters with significant differences in the ANOVA, the Student–Newman–Keuls multiple range test was subsequently applied, and the corresponding groupings are indicated by superscript letters in Table 3 and Table 4.

## 4. Discussion

Clear differences were observed between the group of oleic oils and the two types of reference triglycerides for all characteristic parameters of both models (Table 3 and Table 4); no statistical analysis is needed. Although less pronounced, significative differences were also found within the oleic oil group, for parameters: ε_H_, ε_L_, Δε, Δε_H_ and Δε_L_ as shown by the Student–Newman–Keuls multiple range test (*p* < 0.05), marked in the tables with superscript letters.

The Maxwell model identifies one more independent parameter than the Cole–Cole model, and significantly reduces the PDRMS from 24% to 10%, evidencing a marked improvement in spectral shape fitting. Moreover, the SF values remained similar for both models (3–9%), ruling out the possibility that the improved fit resulted from overfitting of the experimental noise.

Additionally, two further tests were performed in order to compare both models. The reduced chi-squared for the Maxwell model (5 parameters) was lower than for the Cole–Cole model (4 parameters), indicating a marked improvement in the relative spectral fit. The Akaike information criterion (AIC) values were −808.66 for the Cole–Cole model and −806.73 for the Maxwell model, yielding a difference in ΔAIC = 1.93. Since this value is below the commonly accepted threshold of two, both models are statistically equivalent from a statistical–theoretic perspective (Table S4 in the Supplementary Materials).

### 4.1. Parameters to Both: Cole–Cole and Maxwell Models

**Figure 11:**
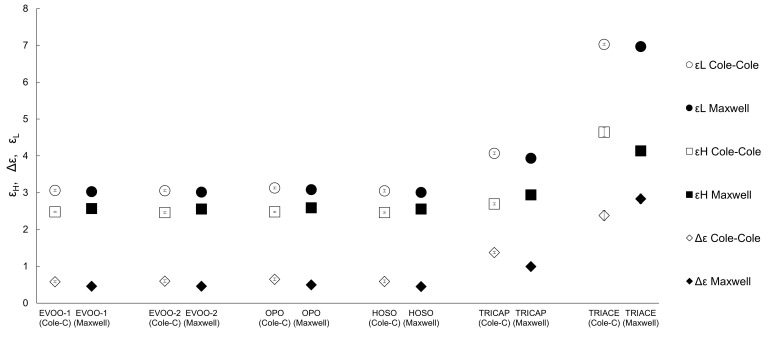
Dielectric constants: ε_L,_ ε_H_, and dielectric increment Δε, for oleic oils and triglycerides, for Cole–Cole and Maxwell models. Error bars are smaller than the symbols and are therefore not visible. Solid line, dot line, short-dashed line and long-dashed line show average reference values for ε_L_ found in the bibliography for extra virgin olive oils (EVOO-1 and EVOO-2), olive pomace oil (OPO), high-oleic sunflower oil (HOSO), tricaprylin (TRICAP), and triacetin (TRIACE), respectively. References in the text.

Figure 11 displays the average of the three common parameters and error bars for oleic oils, tricaprylin, and triacetin, as derived from both models: Δε at the bottom, ε_H_ in the middle, and ε_L_ (the sum of the previous two) at the top.

The values of the common parameters were similar in both models (Figure 11), and also the estimated standard deviation of the weighted residuals (SF < 9%) (Table 3 and Table 4). Nevertheless, the Maxwell model proved to be slightly more accurate than the Cole–Cole model, considering the weighted uncertainty of the five Maxwell fitted parameters (PDRMS < 10%) in comparison with the four Cole–Cole fitted parameters (PDRMS < 24%).

Similar values of the three parameters were observed for the oleic oils, whereas higher values were obtained for tricaprylin and triacetin with both models. The values of ε_L_, Δε, and ε_H_ defined three distinct groups of materials, ordered from highest to lowest as triacetin, tricaprylin, and oleic oils, respectively.

The ANOVA revealed significant differences (*p* < 0.05) for ε_L,_ ε_H_ and Δε between the Cole–Cole and Maxwell models (Supplementary Materials, Table S3), as well as among the different oleic oils within the same model (Supplementary Materials, Tables S1 and S2).

In the Cole–Cole model, ε_L_ of OPO was significantly higher than that of the other oils (Table 3). This result was repeated with the Maxwell model; nevertheless, additional significant differences were found among all oils, which ranked them from the lowest to the highest as HOSO, EVOO-2, EVOO-1, and OPO (Table 4).

Subsequently, a comparative study is carried out between ε_L_ and the ε_s_ reported in the literature for the materials measured in this work.

Triacetin, with three acetate groups (C2:0) attached to a glycerol backbone, is small and compact, resulting in a high density of polar ester groups. Consequently, it exhibits a relatively high ε_L_ = 7.03 (Cole–Cole), ε_L_ = 6.97 (Maxwell), reflecting its pronounced polar character. In the bibliography, ε_s_ = 7.294 (20 °C), ε_s_ = 6.904 (40 °C) [4], an interpolated reference value is depicted in a long-dashed line in Figure 11.

Tricaprylin contains three ester groups; these polar structures are attached to longer, predominantly weakly polar hydrocarbon chains (C8:0). This structural difference dilutes the overall polar density of the molecule. As a result, tricaprylin, as expected, has a significantly lower ε_L_ than triacetin (ε_L_ = 4.07 (Cole–Cole), ε_L_ = 3.93 (Maxwell)). In the literature, values of the static dielectric constant were reported as 3.85 and 3.931 (20 °C) [4,64], represented in short-dashed line in Figure 11, and were in accordance with the values obtained in this work.

Oleic oils consist predominantly of long-chain triglycerides, mainly oleic acid (C18:1)**,** a monounsaturated fatty acid. These oils contain one double bond per fatty acid chain, which contributes additional electronic polarizability. However, their long hydrocarbon chains (18 carbons) dramatically reduce the effect of polar ester groups compared to triacetin and tricaprylin. As a result, oleic oils exhibited the lowest ε_L_ among the measured materials, in the range of ε_L_ = 3.05–3.13 (Cole–Cole), ε_L_ = 3.008–3.084 (Maxwell) for the four types of oleic oils measured at 25 °C. Data reported in the bibliography are consistent with our results, taking into account the extended range covered by the two models. For HOSO ε_L_ = 3.008–3.050 was consistent with ε_s_ = 3.037–3.085 (25 °C) [11,13], represented in Figure 11 as a dotted line. For EVOO (1 and 2) ε_L_ = 3.03–3.06 was within the broad interval ε_s_ = 3.04–3.19 (25–20 °C) obtained by different authors [12,17,19,21,25], the average value is depicted with a solid line in Figure 11. To the best of our knowledge, no static permittivity values were reported for OPO. However, its higher value ε_L_ = 3.084–3.130, compared with the other oils, can be attributed to its MUFA content (approximately 10% in OPO vs. 6% in EVOO and 7% in HOSO, Table 1), which provides additional electronic polarizability due to the presence of double bonds [4].

Based on the previous results, ε_L_ was a good estimate of ε_s_, reflecting differences between oleic oils and triglycerides, mainly due to the length of their fatty acid chains and, within the oleic oils, to differences in the type and degree of saturation of their fatty acids.

The values of ε_H_ and Δε depend on the measurement frequency range; therefore, those reported in this work (Table 3 and Table 4) correspond to the analyzed range *f* = 63–864 MHz.

The calculated ε_H_ values for tricaprylin were 2.70 (Cole–Cole) and 2.94 (Maxwell), whereas for triacetin, the values were 4.65 (Cole–Cole) and 4.14 (Maxwell).

Measured values of ε_H_ in oleic oils were in the broad range between 2.459 and 2.940, for Cole–Cole and Maxwell, respectively. In other studies, the high-frequency dielectric constant, ε_∞_, was indirectly calculated from refractive index measurements (ε_∞_ = n^2^), with characteristic values reported between 2.11 and 2.16 for olive oil [13]. Values measured at 9.1 GHz showed higher: 2.40–2.42 at temperatures of 20–30 °C [19], which were closer to those obtained in the present work for olive oils, measured at lower frequencies.

Regarding the Δε, oleic oils were clearly separated from the two types of triglycerides; furthermore, a significant difference in OPO was also revealed within the oleic oils for both the Cole–Cole and Maxwell models (Table 4 and Figure 11).

### 4.2. Specific Parameters of the Maxwell Model

As with Δε, the dielectric increments Δε_H_ and Δε_L_ differed among the three groups: oleic oils, tricaprylin, and triacetin. For the oleic oils and tricaprylin, the dielectric increment associated with low-frequency relaxation was smaller than that of high-frequency relaxation, whereas triacetin showed the opposite order for these increments.

Among the oils, the dielectric increment most sensitive was Δε_H_, which showed significant differences that classified the type of oil (HOSO 0.269, EVOO 0.278–0.281, and OPO 0.301) as distinct, according to their origin (Table 4). There is no data available in the literature for these parameters.

### 4.3. Specific Parameters of the Cole–Cole Model

The characteristic parameters of the CPE element are: Y_0_ (absolute value of the capacitive factor, expressed as—Log(Y_0_/Y_r_), Y_r_ = 1 (S·s^α^) for homogeneity) and α (capacitive exponent). Linear correlations between α and log(Y_0_/Y_r_) were found from the fifteen measurements carried out for each material, with correlation coefficients R^2^ = 0.98–0.99 and slopes of 0.1454, 0.1180, and 0.1093–0.1126 for triacetin, tricaprylin, and the oleic oils, respectively.

These strong correlations between the two parameters, α and Y_0_ (through its logarithm), indicate that they should not be used as separate and independent parameters to characterize the analyzed materials; only the pair of values taken together (with their respective uncertainties) can be used for this purpose.

**Figure 12:**
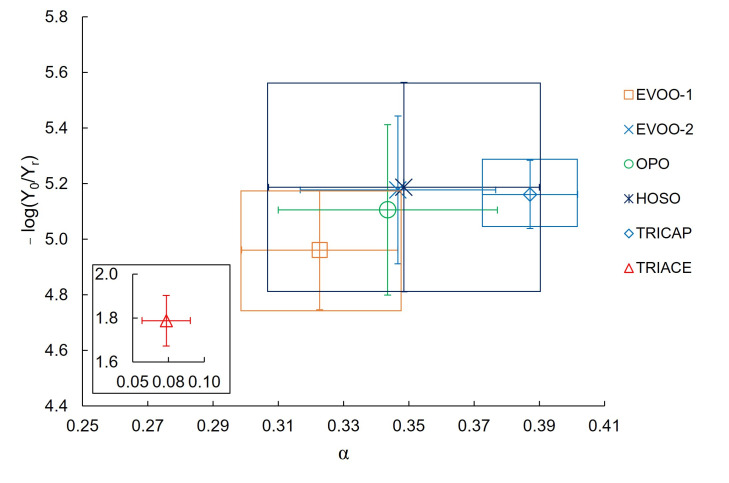
Capacitive factor—Log(Y_0_/Y_r_) vs. capacitive exponent α for extra virgin olive oils (EVOO-1, EVOO-2), olive pomace oil (OPO), high-oleic sunflower oil (HOSO), tricaprylin (TRICAP), and triacetin (TRIACE). For homogeneity, Y_r_ = 1 (S·s^α^). Inset showing triacetin.

In Figure 12, the average values of −log(Y_0_/Y_r_) versus α, with their error rectangles, are shown, where triacetin and tricaprylin can be distinguished from each other and from the oleic oils, while no significant differences were observed among the different oleic oils.

The α parameter was around 0.30–0.40 for the oleic oils, in agreement with the values reported by [21], 0.387 for tricaprylin, and 0.05–0.09 for triacetin. These values of α ≥ 0.2 indicate that the oleic oils and tricaprylin exhibited several simple relaxations in the analyzed frequency interval, whereas triacetin, with α < 0.1, showed a narrower, almost Debye-type relaxation (inset in Figure 12). These results are consistent with triacetin being a polar SCT, whereas the other weakly polar LCT and MCT display broader dielectric relaxations.

According to the literature [65], triacetin presents four separated relaxations in the frequency range of 0.1–100 GHz. The first relaxation is a simple Debye at 350 MHz, the second relaxation at 2500 MHz Cole–Cole, and the following relaxations are very far away from our interval of measurement, at 15 GHz and 50 GHz. Therefore, the first two relaxations reported for triacetin, a Debye process and a weakly broadened Cole–Cole process separated by more than 2000 MHz, were effectively captured, within the frequency range of this study, as a single Cole–Cole relaxation with α = 0.073 ± 0.017. This very low α value reflects a nearly Debye-like behavior and arises from the large separation between the two underlying relaxation frequencies (Table 3).

### 4.4. Characteristic Relaxation Frequencies for Cole–Cole and Maxwell Models

The relaxation processes in the frequency domain were analyzed for both models. Being the central relaxation frequency (for a symmetric relaxation): f_C_ is for the Cole–Cole model, and f_L_ and f_H_ (low- and high-frequency components) are for the Maxwell model.

The frequency f_C_ was obtained from the elements of the Cole–Cole equivalent circuit: C_s_, α, and Y_0_ (Equation (7)). The frequencies f_L_ and f_H_ were calculated from the two time constants (τ_L_, τ_H_) obtained directly from the Maxwell equivalent circuit.

**Figure 13:**
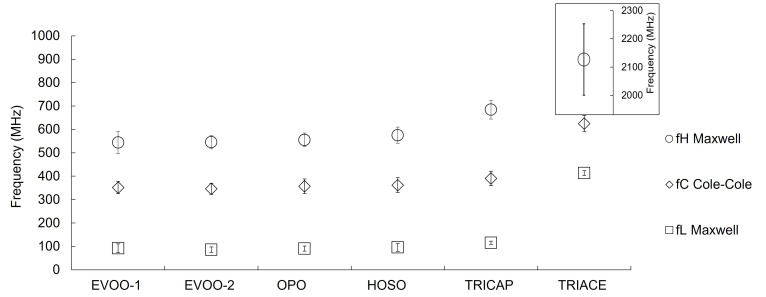
Average characteristic relaxation frequencies: Cole–Cole frequency f_C_ and the Maxwell frequencies, low-frequency f_L_ and high-frequency f_H_. Standard deviations from fifteen measurements for each material are included. f_H_ for triacetin is shown in the upper-right inset.

Figure 13 shows the three frequencies for all oleic oils and triglycerides, in the order f_L_, f_C_, and f_H_ (in MHz).

In Figure 13, f_C_ lies between the two Debye relaxation frequencies, f_L_ and f_H_. The uncertainties for all these frequencies of each triglyceride and oleic oil were less than 9% for f_C_, less than 12% for f_H_, and less than 24% for f_L_, which showed the highest uncertainty.

The Cole–Cole frequency values of triacetin derived from the Cole–Cole model (f_C_ = 625 ± 35 MHz) were higher than the first Debye relaxation frequency reported in the literature. To the best of our knowledge, no Cole–Cole parameter values for triacetin have been reported in the literature. The low-frequency (f_L_ = 414 MHz) and high-frequency (f_H_ = 2127 MHz) relaxation frequencies determined for triacetin (Table 4) exhibited low uncertainty (< 6%) and are consistent with the values of 350 MHz and 2500 MHz at 20 °C reported by [65]. This excellent agreement thus confirms the reliability of our methodology—encompassing the measurement, data transformation, and subsequent analysis—for the identification of two elementary Debye relaxations via the Maxwell model over the frequency range covered in this work.

The fc values obtained for the oleic oils, ranging from 324 to 391 MHz, were consistent with the reference data: 240–340 MHz for olive oils [21,25]. To the best of our knowledge, no reference data are available for tricaprylin.

The f_H_ clearly distinguished the three groups: oleic oils (ranging from 544 to 576 MHz), tricaprylin (685 ± 40 MHz), and triacetin (2127 ± 126 MHz) (Table 4 and Figure 13). However, when the oleic oils were analyzed separately, no significant differences were found among them. Hence, the high-frequency relaxation can be considered a parameter strongly influenced by the fatty acid chain length of the triglycerides constituting each sample. There is no reference to this relaxation for oleic oils nor tricaprylin; therefore, it is an important question to analyze in future work.

The f_L_ results were relevant, as they appeared within the same range for all the measured weakly polar materials. For the oleic oils, f_L_ = 92 ± 18, 86 ± 12, 90 ± 11, and 96 ± 17 MHz, for EVOO-1, EVOO-2, OPO, and HOSO, respectively. These values were of the same order of magnitude as those found for tricaprylin, f_L_ = (116 ± 7 MHz). In contrast, no relaxation frequency close to 100 MHz was observed for triacetin within these ranges, but the higher low-frequency f_L_ values (404–424 MHz) found for triacetin are likely due to its higher polarity.

Thus, f_L_ values observed for all oleic oils (86–96 MHz) and tricaprylin (114 MHz) were consistent with the main glycerol relaxation at 25 °C (100 MHz), which Zhou et al. described as the major contributor to microwave heating of vegetable oils [26,27].

## 5. Conclusions

The behavior of OECP-5p was verified as an antenna, with L = 4 mm and z ≈ 0.63, for the reference dielectric compounds water and acetone, and z ≈ 0.47 for oleic oils. The measurement procedure, together with the Marsland transformation, was validated using silicone oil, with a null loss dielectric factor, and glycerol with a relaxation frequency of 114 MHz. The low-frequency dielectric constants (ε_L_) obtained with both the Cole–Cole and Maxwell models were consistent with the static dielectric constants reported in the literature. The results reflected differences between oleic oils and triglycerides, mainly due to the length of the fatty acid chains.

The Maxwell model provided better statistical fits than the Cole–Cole model and revealed significant differences among all the oleic. The two relaxations, at the low-frequency f_L_ = 414 MHz and the high-frequency f_H_ = 2127 MHz, found for triacetin, validated the proposed method for identifying two simple Debye relaxations through the Maxwell model. The low-frequency, f_L_, was particularly relevant, as it appeared in the same range (86–96 MHz) for all oleic oils, and (99–123 MHz) for tricaprylin, close to the relaxation frequency of glycerol. This result is consistent with recent studies showing that the glycerol backbone of triglycerides is a major contributor to the efficient microwave heating of vegetable oils. Additionally, the low-cost equipment and the open-source software make the method accessible to laboratories with limited resources.

As future work, it is imperative to characterize both the low- and high-frequency relaxations as a function of temperature and microwave irradiation.

## Figures and Tables

**Table 1:** Distribution of the principal fatty acids in the oleic oils, as provided by the manufacturers.

Oleic Oil	MUFA (%)	PUFA (%)	SFA (%)	(MUFA + PUFA)/SFA
EVOO-1	74	7	19	4.3
EVOO-2	81	6	13	6.7
OPO	75	10	15	5.7
HOSO	84	7	9	10.1

EVOO-1 and EVOO-2: extra virgin olive oil from Cuenca and Jaén, respectively; OPO: olive pomace oil from Jaen; HOSO: high-oleic sunflower oil. Fatty acids percentages: MUFA (%): monounsaturated fatty acids; PUFA (%): polyunsaturated fatty acids; SFA (%): saturated fatty acids. Free fatty acid content was below 0.4%.

**Table 2:** Characteristic parameter z of the Clarkson antenna radiative theory of the OECP-5p for the measured compounds.

OECP-5p	Re(S_11_) Fitting	Im(S_11_) Fitting		Real Fitting Re(S_11_)_estim_ = m·Re(S_11_)_exp_ + n			Imaginary Fitting Im(S_11_)_estim_ = a·Im(S_11_)_exp_ + b		
Compounds	z	z	ε_s_	m	n	R^2^	a	b	R^2^
Water	0.613	0.635	80	0.9952	−0.015	0.9970	1.028	0.0188	0.9999
Acetone	0.621	0.625	20	1.009	0.0069	0.9997	1.010	0.0060	0.9998
Triacetin	0.594	0.561	7.0 *	1.007	−0.0061	0.9994	1.020	0.0058	0.9996
Tricaprylin	0.555	0.495	4.1 *	1.017	0.0163	0.9970	1.022	0.0033	0.9996
Silicone oil	0.521	0.505	2.8	0.975	0.0025	0.9700	1.001	0.0005	0.9995
EVOO-1	0.673	0.466	3.1 *	1.120	−0.1180	0.9780	1.010	0.0013	0.9993
EVOO-2	0.516	0.473	3.1 *	1.000	0.0006	0.9810	1.009	0.0012	0.9992
OPO	0.525	0.472	3.1 *	1.015	−0.0140	0.9910	1.008	0.0010	0.9996
HOSO	0.518	0.470	3.1 *	1.012	0.0110	0.9910	1.011	0.0010	0.9995
Air	0.129	0.154	1.0	−	−	−	0.950	−0.0010	0.9636

OECP-5p: open-ended coaxial probe of five pins; EVOO-1 and EVOO-2: extra virgin olive oils; OPO: olive pomace oil; HOSO: high-oleic sunflower oil; ε_s_: relative dielectric constant; *: ε_L_ from Cole–Cole fits. Linear fitting coefficients (m, n for Re(S_11_) and a, b for Im(S_11_)) and determination coefficients (R^2^) for each compound.

**Table 3:** Average Cole–Cole parameters for oleic oils and triglycerides.

	ε_H_	−Log(Y_0_/Yr)	α	f_C_ (MHz)	Δε	ε_L_	PDRMS (%)	SF (%)
EVOO-1	2.483 ± 0.013 ^b^	4.96 ± 0.21	0.323 ± 0.024	352 ± 26	0.58 ± 0.03 ^a^	3.06 ± 0.03 ^a^	13–19	6–8
EVOO-2	2.459 ± 0.022 ^a^	5.18 ± 0.27	0.35 ± 0.03	346 ± 23	0.60 ± 0.04 ^a^	3.06 ± 0.02 ^a^	16–24	8–9
OPO	2.481 ± 0.01 ^b^	5.1 ± 0.3	0.34 ± 0.03	360 ± 30	0.65 ± 0.04 ^b^	3.13 ± 0.03 ^b^	12–19	7–9
HOSO	2.461 ± 0.017 ^a^	5.2 ± 0.4	0.35 ± 0.04	360 ± 30	0.59 ± 0.04 ^a^	3.05 ± 0.03 ^a^	15–23	4–9
TRICAP	2.70 ± 0.03	5.16 ± 0.12	0.387 ± 0.015	390 ± 30	1.37 ± 0.03	4.07 ± 0.04	6–8	3–4
TRIACE	4.65 ± 0.13	1.79 ± 0.12	0.073 ± 0.017	630 ± 40	2.38 ± 0.14	7.03 ± 0.03	2–4	3–4

EVOO: extra virgin olive oil; OPO: olive pomace oil; HOSO: high-oleic sunflower oil; TRICAP: tricaprylin; TRIACE: triacetin; ε_H_: high-frequency dielectric constant; −log(Y_0_/Y_r_): absolute value of the logarithm of the capacitive factor; α: capacitive exponent; f_C_: Cole–Cole relaxation frequency; Δε: dielectric increment; ε_L_: low-frequency dielectric constant. PDRMS: the root-mean-square of the relative residuals, and SF: standard deviation of the weighted residuals of the model, both parameters are reported as interval values of fifteen fittings. Different superscript letters indicate significant differences (*p* < 0.05) between oleic oils; significance was observed only for ε_H_, Δε and ε_L_.

**Table 4:** Average Maxwell parameters for oleic oils and triglycerides.

	ε_H_	Δε_H_	Δε_L_	f_L_ (MHZ)	f_H_ (MHZ)	Δε	ε_L_	PDRMS (%)	SF (%)
EVOO-1	2.571 ± 0.017 ^a^	0.281 ± 0.018 ^b^	0.178 ± 0.011 ^a^	92 ± 18	540 ± 50	0.459 ± 0.024 ^a^	3.03 ± 0.03 ^b^	4–5	6–8
EVOO-2	2.556 ± 0.014 ^a^	0.278 ± 0.009 ^b^	0.181 ± 0.014 ^a^	86 ± 12	545 ± 28	0.460 ± 0.015 ^a^	3.016 ± 0.017 ^ab^	4–5	7–9
OPO	2.589 ± 0.017 ^b^	0.301 ± 0.011 ^c^	0.194 ± 0.018 ^b^	90 ± 11	555 ± 29	0.496 ± 0.017 ^b^	3.084 ± 0.019 ^c^	4–10	7–9
HOSO	2.558 ± 0.020 ^a^	0.269 ± 0.011 ^a^	0.180 ± 0.012 ^a^	96 ± 17	580 ± 30	0.449 ± 0.018 ^a^	3.008 ± 0.026 ^a^	4–7	7–9
TRICAP	2.940 ± 0.018	0.550 ± 0.012	0.445 ± 0.011	116 ± 7	685 ± 40	0.995 ± 0.018	3.93 ± 0.03	1–2	4–5
TRIACE	4.14 ± 0.13	1.39 ± 0.17	1.45 ± 0.04	414 ± 10	2130 ± 130	2.83 ± 0.13	6.97 ± 0.03	1–6	3–4

EVOO: extra virgin olive oil; OPO: olive pomace oil; HOSO: high-oleic sunflower oil; TRICAP: tricaprylin; TRIACE: triacetin; ε_H_: high-frequency dielectric constant; Δε_H:_ high-frequency dielectric increment; Δε_L_: low-frequency dielectric increment; f_L_: low-frequency relaxation frequency; f_H_: high-frequency relaxation frequency; Δε: dielectric increment; ε_L_: low-frequency dielectric constant. PDRMS: the root-mean-square of the relative residuals, and SF: standard deviation of the weighted residuals of the model, both parameters are reported as interval values of fifteen fittings. Different superscript letters indicate significant differences (*p* < 0.05) between oleic oils; significance was observed only for ε_H_, Δε_H_, Δε_L,_ Δε and ε_L_.

## Data Availability

The original contributions presented in the study are included in the article/Supplementary Material, further inquiries can be directed to the corresponding author.

## Supplementary Materials

The following supporting information can be downloaded at: https://www.mdpi.com/article/10.3390/foods15142460/s1, Table S1: ANOVA table with mean squares (MS), degrees of freedom (df), F-ratio and P-value for the effect of the oils on the Cole−Cole model parameters: high-frequency dielectric constant (ε_H_), absolute value of the logarithm of capacitive factor (−Log(Y_0_)), capacitive exponent (α), relaxation frequency (f_C_), dielectric increment (Δε) and low-frequency dielectric constant (ε_L_); Table S2: ANOVA table with mean squares (MS), degrees of freedom (df), F-ratio and P-value for the effect of the oils on the Maxwell model parameters: high-frequency dielectric constant (ε_H_), high-frequency dielectric increment (Δε_H_), low-frequency dielectric increment (Δε_H_), relaxation frequency at low frequencies (f_L_), relaxation frequency at high frequencies (f_H_), total frequency dielectric increment (Δε= Δε_H_ + Δε_L_), low-frequency dielectric constant (ε_L_); Table S3:ANOVA table with mean squares (MS), degrees of freedom (df), F-ratio and P-value for the effect of the type of model for the common parameters, high-frequency dielectric constant (ε_H_), dielectric increment (Δε) and low-frequency dielectric constant (ε_L_); Table S4: Comparative summary of goodness-of -fit metrics. Number of experimental data points (N), number of adjustable parameters in each model (p), degrees of fredoom (df), percentage root-mean-square of the relative residuals (PDRMS), standard deviation of the weighted residuals (SF), weighted sum of squares (S), reduced chi-squared, Akaike information criterion (AIC)..

## Abbreviations

The following abbreviations are used in this manuscript:

OECP-5p	Open-ended coaxial probe with 5 pins
EVOO	Extra virgin olive oil
OPO	Olive pomace oil
HOSO	High-oleic sunflower oil
TRICAP	Tricaprylin
TRIACE	Triacetin
LCT	Long-chain triglyceride
MCT	Medium-chain triglyceride
SCT	Short-chain triglyceride
SFA	Saturated fatty acids
MUFA	Monounsaturated fatty acids
PUFA	Polyunsaturated fatty acids
PDRMS	Root-mean-square value of the relative residuals of all free parameters of the fit
SF	Estimated standard deviation of the weighted residuals
