# Supplementary material for: Identification of Two Dielectric Relaxations in Oleic-Rich Oils Within the 50–900 MHz Range Using a Low-Cost Method
Source: Foods. 2026 Jul 11;15(14):2460. doi: 10.3390/foods15142460 (PMC13409024; doi:10.3390/foods15142460)
Supplement: Supplementary file 1 [file foods-15-02460-s001.zip › foods-4368974-supplementary.pdf]

Table S1. ANOVA table with mean squares (MS), degrees of freedom (df), F-ratio and P-value for the effect of the oils on the Cole-Cole model parameters: high-frequency dielectric constant (ε_H), absolute value of the logarithm of capacitive factor (-Log(Y_0)), capacitive exponent (α), relaxation frequency (f_C), dielectric increment ($\Delta\varepsilon$) and low-frequency dielectric constant (ε_L).

		MS					
	**df (-)**	**ε_H (-)**	**-Log(Y_0) (-)**	**α (-)**	**f_C (MHz)^2**	**$\Delta\varepsilon$ (-)**	**ε_L (-)**
**Oil**	3	0.0024327	0.163594	0.00214	0.00214	0.0128106	0.0190189
**Error**	56	0.000326502	0.0881351	0.00108016	0.00108016	0.00129963	0.000852854
	**F-ratio**	7.45	1.86	1.98	1.98	9.86	22.3
	**P-value**	0.0003	0.1475	0.1272	0.1272	0.0000	0.0000

Table S2. ANOVA table with mean squares (MS), degrees of freedom (df), F-ratio and P-value for the effect of the oils on the Maxwell model parameters: high-frequency dielectric constant (ε_H), high-frequency dielectric increment ($\Delta\varepsilon_H$), low-frequency dielectric increment ($\Delta\varepsilon_H$), relaxation frequency at low frequencies (f_L), relaxation frequency at high frequencies (f_H), total frequency dielectric increment ($\Delta\varepsilon = \Delta\varepsilon_H + \Delta\varepsilon_L$), low-frequency dielectric constant (ε_L).

		MS						
	**df (-)**	**ε_H (-)**	**$\Delta\varepsilon_H$ (-)**	**$\Delta\varepsilon_L$ (-)**	**f_L (MHz)^2**	**f_H (MHz)^2**	**$\Delta\varepsilon$ (-)**	**ε_L (-)**
**Oil**	3	0.00330989	0.00274444	0.000829885	237.601	3172.73	0.00624687	0.0178361
**Error**	56	0.000288206	0.000159817	0.000189471	217.78	1275.69	0.000357398	0.000588445
	**F-ratio**	11.48	17.17	4.38	1.09	2.49	17.48	30.31
	**P-value**	0.0000	0.0000	0.0077	0.3605	0.0698	0.0000	0.0000

Table S3. ANOVA table with mean squares (MS), degrees of freedom (df), F-ratio and P-value for the effect of the type of model for the common parameters, high-frequency dielectric constant (ε_H), dielectric increment ($\Delta\varepsilon$) and low-frequency dielectric constant (ε_L).

		MS		
	**df (-)**	**ε_H (-)**	**$\Delta\varepsilon$ (-)**	**ε_L (-)**
**Model**	1	0.286114	0.566433	0.047402
**Error**	118	0.000437725	0.0012709	0.00162
	**F-ratio**	653.64	445.70	29.24
	**P-value**	0.0000	0.0000	0.0000

Table S4. Comparative summary of goodness-of -fit metrics. Number of experimental data points (N), number of adjustable parameters in each model (p), degrees of fredoom (df), percentage root-mean-square of the relative residuals (PDRMS), standard deviation of the weighted residuals (SF), weighted sum of squares (S), reduced chi-squared (χ^2_{red}), Akaike information criterion (AIC).

Model	Physical description	N	Parameters (p)	df (N-p)	PDRMS (%)	SF (%)	S	χ^2_{red}	AIC
Cole-Cole	One distributed relaxation	180	4	176	24	9	1.92875	0.0589	-808.66
Maxwell	Two Debye relaxations	180	5	175	10	9	1.92837	0.0103	-806.73

χ^2_{red} = (PDRMS / 100)^2·N / (N - p) and AIC = N·ln (S / N)+ 2p
